# Involvement of the Integrin α1β1 in the Progression of Colorectal Cancer

**DOI:** 10.3390/cancers9080096

**Published:** 2017-07-26

**Authors:** Salah Boudjadi, Gérald Bernatchez, Blanche Sénicourt, Marco Beauséjour, Pierre H. Vachon, Julie C. Carrier, Jean-François Beaulieu

**Affiliations:** 1 Laboratory of Intestinal Physiopathology, Department of Anatomy and Cell Biology, Faculty of Medicine and Health Sciences, Université de Sherbrooke, Sherbrooke, QC J1H 5N4, Canada; salah.boudjadi@usherbrooke.ca (S.B.); blanche.senicourt@usherbrooke.ca (B.S.); marco.beausejour@usherbrooke.ca (M.B.); pierre.h.vachon@usherbrooke.ca (P.H.V.); 2 Department of Medicine, Faculty of Medicine and Health Sciences, Université de Sherbrooke, Sherbrooke, QC J1E 4K8, Canada; gerald.bernatchez@usherbrooke.ca (G.B.); julie.carrier2@usherbrooke.ca (J.C.C.)

**Keywords:** ITGA1, integrin α1, proliferation, survival, colorectal cancer, cell migration

## Abstract

Integrins are a family of heterodimeric glycoproteins involved in bidirectional cell signaling that participate in the regulation of cell shape, adhesion, migration, survival and proliferation. The integrin α1β1 is known to be involved in RAS/ERK proliferative pathway activation and plays an important role in fibroblast proliferation. In the small intestine, the integrin α1 subunit is present in the crypt proliferative compartment and absent in the villus. We have recently shown that the integrin α1 protein and transcript (*ITGA1*) are present in a large proportion of colorectal cancers (CRC) and that their expression is controlled by the MYC oncogenic factor. Considering that α1 subunit/*ITGA1* expression is correlated with MYC in more than 70% of colon adenocarcinomas, we postulated that the integrin α1β1 has a pro-tumoral contribution to CRC. In HT29, T84 and SW480 CRC cells, α1 subunit/*ITGA1* knockdown resulted in a reduction of cell proliferation associated with an impaired resistance to anoikis and an altered cell migration in HT29 and T84 cells. Moreover, tumor development in xenografts was reduced in HT29 and T84 sh-ITGA1 cells, associated with extensive necrosis, a low mitotic index and a reduced number of blood vessels. Our results show that α1β1 is involved in tumor cell proliferation, survival and migration. This finding suggests that α1β1 contributes to CRC progression.

## 1. Introduction

As a unique family of heterodimeric transmembrane glycoproteins, integrins are characterized by their involvement in bidirectional cell signaling that participates in the regulation of cell shape, adhesion, migration, survival and proliferation. To date, 8 β and 18 α subunits have been identified, which form 24 different integrins distinguished by their expression pattern, localization and role under physiological and pathological conditions. The integrin α1β1 is known to be involved in the mitogen-activated protein kinase (MEK [MAPK] kinase)/extracellular regulated kinase (ERK) proliferative pathway activation through the recruitment of caveolin-1 and the Src homology 2 domain containing transforming protein 1 (Shc) by the integrin α1 subunit [1,2,3]. In addition, α1 contains an “I” domain involved in α1β1 binding to collagen [1,4,5]. Furthermore, it was demonstrated that the integrin α1β1 regulates different pathways and functions through specific amino acids in the cytoplasmic tail of the integrin α6 subunit [6]. These characteristics reflect the reported involvement of the integrin α1β1 in the proliferation of fibroblasts, endothelial cells and in mammary carcinoma cell proliferation and migration [4,6,7,8]. In colorectal cancer (CRC), a few studies have shown that α1β1 regulates CRC cell invasion through association with talin and paxillin and the activation of the Crk-associated substrate (p130Cas)/c-Jun cascade [9]. CRC, one of the leading causes of death in western countries [10], is well known to be a multi-step process that involves successive mutation, epigenetic alteration and gene dysregulation, leading to increased cell proliferation, tissue invasion and metastasis. We have previously shown that the integrin α1 subunit/*ITGA1* is present in the crypt proliferative compartment and normally absent in the villus of the small intestine and in the surface epithelium of the colon [11]**,** while in CRC, it is present in 65% of tumors where its expression appears to be regulated by the oncogenic MYC transcription factor [12], suggesting that the integrin α1β1 is involved in colorectal neoplasia. In this study, we have investigated this possibility. We demonstrate that the integrin α1β1 is involved in the proliferation, migration and survival of CRC cells, supporting a role for this receptor in CRC progression.

## 2. Results

### 2.1. Integrin α1 Subunit/ITGA1 Knockdown in CRC Cells

**Figure 1:**
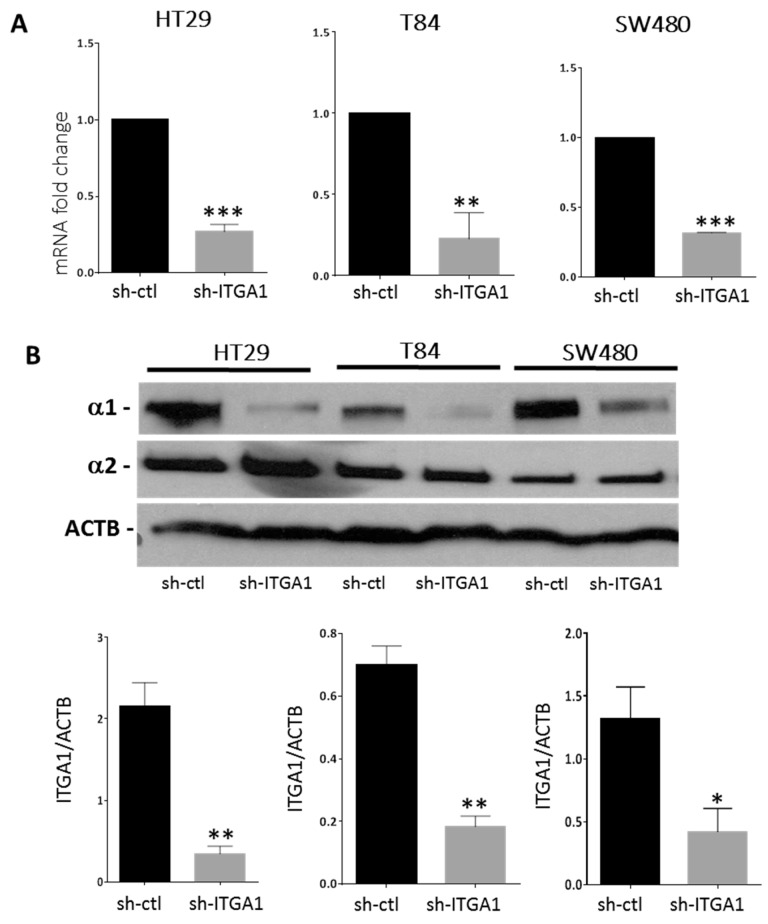
Downregulation of the α1 integrin subunit in colorectal cancer cells. HT29, T84 and SW480 cells were infected with lentiviruses encoding a non-targeting short hairpin RNA (sh-ctl) or with shRNA targeting the *ITGA1* mRNA (sh-ITGA1). Cells were selected with puromycin (10 μg/mL) 10 days before protein or RNA extraction. (**A**) Expression of the transcript of the ITGA1 gene was quantified by qPCR and normalized to the expression of the endogenous gene RPLP0. (**B**) Representative Western blot showing expression of the integrin α1 and α2 subunits in sh-ITGA1 cells compared to sh-ctl cells and densitometric analysis of the α1 subunit. Expression of ACTB was used as the protein loading control. Student’s *t* test. * *p* < 0.05, ** *p* < 0.01, *** *p* < 0.001.

To investigate the involvement of α1β1 in the progression of CRC, we selected the three CRC cell lines HT29, SW480 and T84 expressing the integrin α1 subunit at significant protein levels and opted for a loss of function strategy to study integrin α1β1 involvement in CRC. Knocking down of integrin α1 subunit/*ITGA1* expression was performed using an sh-RNA integrin α1 subunit targeting strategy and was validated at both the transcript and protein levels relative to control sh (sh-ctl vs. sh-ITGA1, Figure 1A,B). The loss of α1β1 did not induce a significant increase in the expression of the integrin α2β1, another collagen receptor present in colorectal cell lines [13], as observed at the protein level in the three cell lines where the integrin α2 subunit remained stable in sh-ITGA1 cells (Figure 1B).

### 2.2. Integrin α1β1 Regulates Proliferation, Anoikis and Migration in CRC Cells

**Figure 2:**
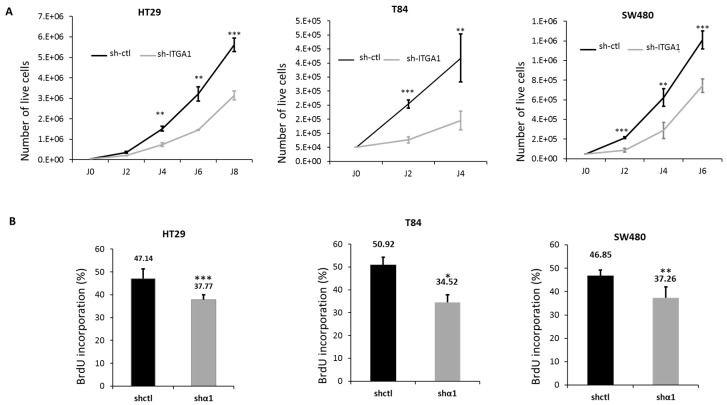
Involvement of the integrin α1β1 in the proliferation of colorectal cancer cells. (**A**) Growth curves showing the cell counts up to 8 days after seeding of HT29, T84 and SW480. The curves show the number of live cells in the two groups; sh-ctl (black) and sh-ITGA1 (gray). Initially, 50000 cells were seeded into 6-well plates, and cells were counted every two days. (**B**) Histogram showing the results of 5-bromo-2′-deoxyuridine (BrdU) incorporation into the cells, performed 4 days after seeding of the same three cell lines. In each field, labeled nuclei were counted and compared to the total number of nuclei stained with 4′,6-diamidino-2-phenylindole (DAPI). The experiments were performed in triplicate and were repeated three times. Student’s *t* test. * *p* < 0.05, ** *p* < 0.01, *** *p* < 0.001.

Since integrin α1β1 was shown to be involved in the proliferation of different cell types including endothelial cells [14], fibroblasts [15] and pulmonary carcinomatous cells [16], we first tested whether this integrin is important for the proliferation of CRC cells. We observed that up to 8 days after cell seeding, there was a significant decrease in cell number for sh-ITGA1 cells compared to sh-ctl cells for the three lines HT29, T84 and SW480 (Figure 2A). A significant reduction in HT29 cell proliferation was also observed with another sh-ITGA1 sequence B (see M&M) in preliminary experiments. The apparent reduction in cell growth of the *ITGA1* knockdown cells was confirmed by a significant reduction in 5-bromo-2′-deoxyuridine (BrdU) incorporation into sh-ITGA1 cells relative to sh-ctl cells for the three cell lines (Figure 2B). These results indicate that the integrin α1β1 is important for the proliferation of colorectal cancer cells.

**Figure 3:**
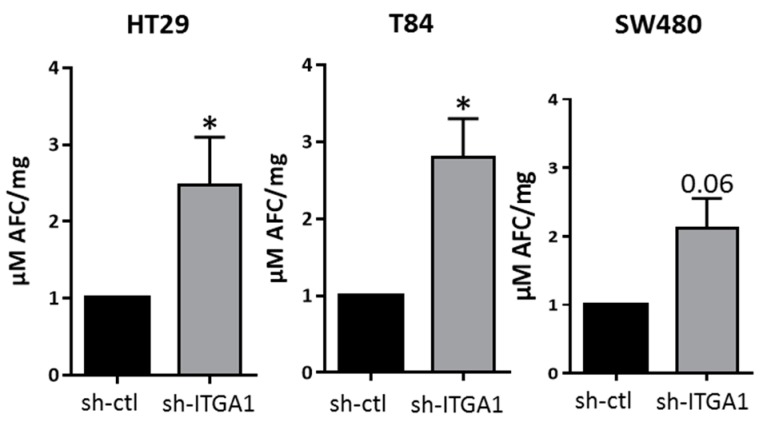
Involvement of integrin α1β1 in anoikis resistance in colorectal cancer. Histograms showing the results of the measurement of caspase-3 activity in the three cell lines HT29, T84 and SW480. The cells were kept in suspension without serum for 24 h in plates coated with poly 2-hydroxyethyl methacrylate. Cells were lysed using buffer without p-nitrophenyl phosphate. N = 3. Student’s *t* test. * *p* < 0.05.

Knowing that integrins participate in the mechanism of cell survival, which is important for cancer cell metastasis [17,18,19], we also evaluated the importance of the integrin α1β1 for this function by measuring caspase-3 activity as an indicator of the activation of irreversible apoptosis [20]. Downregulation of integrin α1 subunit/*ITGA1* expression had no significant effect on apoptosis in adherent HT29, T84 and SW480 cells but a significant increase in caspase-3 activity was observed in the three sh-ITGA1 cell lines kept in suspension (Figure 3), demonstrating that the α1β1 integrin may exert a preventive effect on anoikis in CRC cells.

**Figure 4:**
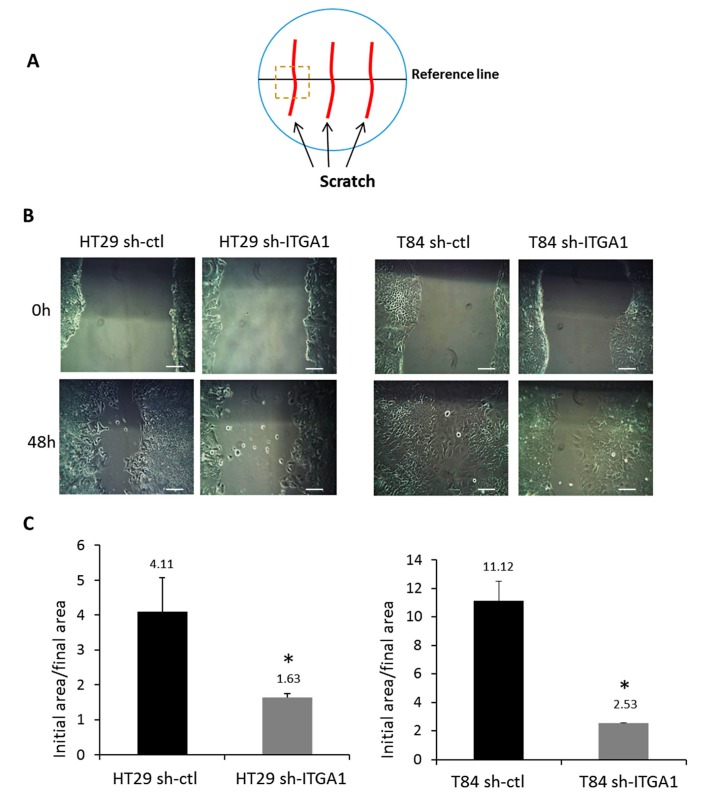
Involvement of the integrin α1β1 in cell migration. (**A**) Schematic representation of the scratch assay procedure. Cells were plated at high density and treated with 2 mM hydroxyurea for 24 h before and throughout the assay. Two to three wounds were made in each Petri dish (red lines). A line (in black) was drawn under the Petri dish to serve as a landmark to track cell migration. (**B**) Representative micrographs of the scratch assay for HT29 and T84 cell lines. The evolution of the closure of the wounded area was compared between sh-ITGA1 and sh-ctl groups from 0 to 48 h. (**C**) Histogram showing the result of the initial area created by the injury (0 h) with respect to the final surface (48 h). The experiments were performed in triplicate and were repeated three times. Student’s *t* test. * *p* < 0.05. Scale bars = 100 μm.

The role of α1β1 in cell migration was evaluated using the scratch test assay on HT29 and T84 cell lines (Figure 4). To prevent any proliferation-related events in the assay, cells were treated with hydroxyurea 24 h prior to the scratch assay and throughout the test. As previously reported, SW480 cells are sensitive to hydroxyurea and thus were excluded from this assay [21]. As illustrated in Figure 4, repression of integrin α1 subunit/ITGA1 expression results in delayed wound healing compared to the control for both HT29 cells (more than two times) and T84 cells (more than 4 times) (Figure 4C). This result reflects an impaired wound closure in sh-ITGA1 cells, suggesting that the integrin α1β1 is important for the migration of CRC cells.

### 2.3. Integrin α1β1 Promotes Tumor Growth

**Figure 5:**
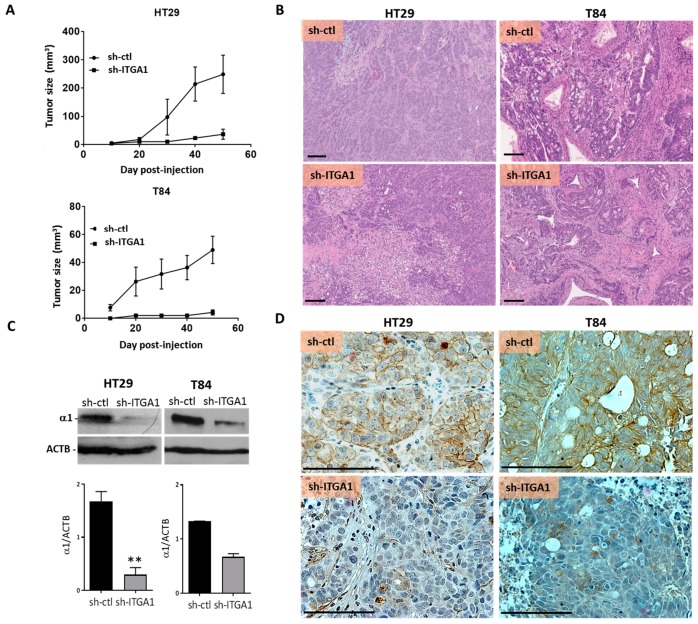
Integrin α1 subunit/*ITGA1* knockdown reduced the development of colorectal tumors in xenografts. (**A**) Tumor growth assessment after injection of 2 × 10^6^ HT29 or T84 cells into the subcutaneous tissue of nu/nu mice. For both cell lines, the sh-ctl and sh-ITGA1 cells were injected after 10 days of selection with puromycin (10 µg/mL). Tumors were measured in two axes and the volume was determined using the formula V = (D × d2)/2. (**B**) Representative micrographs of the histological architecture of the tumors derived from sh-ITGA1 and sh-ctl cells for HT29 and T84 cell lines. Hematoxylin and eosin staining (H&E). (**C**,**D**) Representative Western blot and immunohistochemical micrographs showing the validation of the repression of expression of the α1 subunit, after resection of tumors derived from sh-ctl and sh-ITGA1 cells, for HT29 and T84 cell lines. N = 3. Student’s *t* test. * *p* < 0.05, ** *p* < 0.01, *** *p* < 0.001. Scale bar = 100 µm.

**Figure 6:**
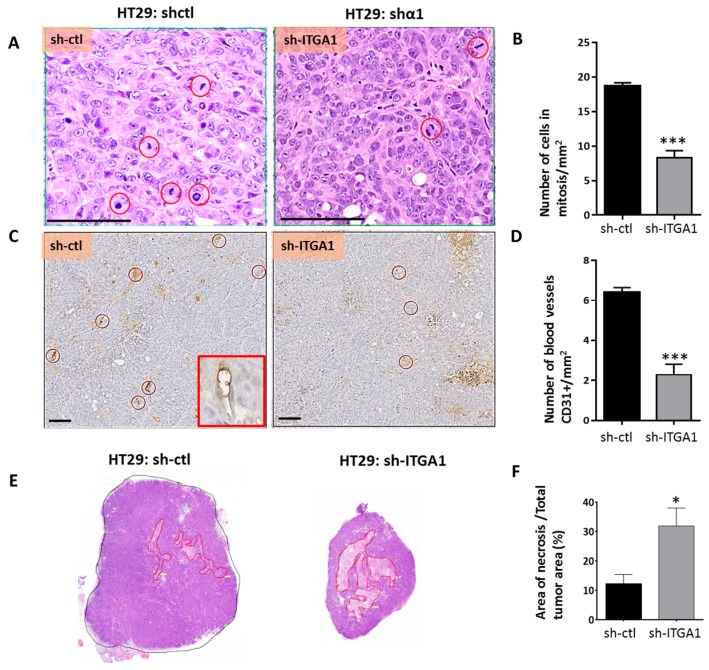
Suppression of the expression of the integrin α1 subunit/*ITGA1* results in fewer mitotic cells, fewer blood vessels and increased necrosis in xenografts. (**A**) Representative micrograph of tumor sections from HT29 sh-ctl and sh-ITGA1 cells stained with H&E. Mitotic cells are surrounded by a red circle. (**B**) Histogram showing the number of mitotic cells per mm^2^ in both sh-ctl and sh-ITGA1 tumors. For each tumor, four areas were randomly selected and analyzed for mitotic count. (**C**) Representative immunohistochemical staining showing intratumoral capillaries surrounded by a red circle. Endothelial cells were identified using anti-CD31 antibodies as shown in the red box. (**D**) Results of the count of the number of capillaries in the sh-ctl and sh-ITGA1 tumors. (**E**) Representative micrograph of sh-ctl and sh-ITGA1 HT29-derived tumors. Tumors were cut along the longest axis and then sections were stained with H&E. For each tumor, the necrotic eosinophilic areas are outlined in red. (**F**) Histogram showing the result of the percentage of necrotic areas compared to the total area of each tumor. The results represent the average analysis of three samples. Student’s *t* test. * *p* < 0.05, *** *p* < 0.001. Scale bar = 100 µm.

Xenografts were used to investigate the involvement of the integrin α1β1 in the tumorigenic capacity of CRC cells. An equivalent number of sh-ITGA1 and sh-ctl cells for both T84 and HT29 cell lines were injected subcutaneously into immunodeficient nu/nu mice. For both HT29 and T84 lines, the decrease in cell proliferation observed in vitro was reflected in vivo by smaller tumors derived from sh-ITGA1 cells compared to the tumors developed from sh-ctl cells (Figure 5A). After tumor excision, morphological analysis showed that downregulation of integrin α1 subunit/*ITGA1* expression did not affect the morphology of HT29 tumor cells, whereas for the T84 line, the sh-ITGA1 cells were smaller in size than the sh-ctl cells, which had a cylindrical shape (Figure 5B). For both HT29 and T84 derived tumors, the maintenance of the downregulation of α1 subunit/*ITGA1* expression was validated using immunohistochemistry and immunoblotting (Figure 5C,D). Histological examination of tumors developed from HT29 cells showed that the number of mitoses in tumor cells was significantly lower in sh-ITGA1 tumors compared to sh-ctl tumors (Figure 6A,B), which is consistent with the decrease in proliferation observed in vitro. In addition, HT29 sh-ITGA1 tumors were found to contain significantly fewer blood vessels detected by CD31 staining than tumors developed from HT29 sh-ctl cells (Figure 6C,D). Related to the paucity of vessels, the sh-ITGA1 tumors showed extended areas of necrosis, which were significantly larger than the necrotic area in sh-ctl tumors (Figure 6E,F). This is clear-cut necrosis, typically observed in ischemic areas. Together, these findings could in part explain the small size of sh-ITGA1 tumors relative to the control tumors and suggest a role for the α1β1 integrin in the progression of colorectal cancer.

## 3. Discussion

In this study, we demonstrated that the α1β1 integrin is required for the proliferation, survival and migration of human colorectal cancer cells. It is well established that integrins, although without intrinsic enzymatic activity, can drive inside-out and outside-in signaling [22]. Depending on the integrin involved, they can modulate several signaling pathways and regulate cell functions including differentiation, gene transcription, survival and proliferation [23,24,25]. In fact, the α1β1 integrin is important for the proliferation of fibroblasts, endothelial cells and lung cancer cells in the murine model [4,6,16]. Indeed, α1β1 recruits caveolin-1 via the α1 subunit, which in turn binds to adaptive molecules such as the FYN oncogene related to SRC (Fyn)/v-yes-1 Yamaguchi sarcoma viral oncogene homolog 1 (Yes) and the growth factor receptor-bound protein 2 (Grb2) [4,5] leading to the activation of the RAS/MEK/ERK pathway [15]. Grb2 is expressed in colorectal cancer [26], and is important in its carcinogenesis [27], while caveolin-1 is positively regulated via the RAS/MEK/ERK pathway in this cancer [28]. It is therefore possible that the integrin α1β1 regulates cell proliferation via the caveolin-1/Grb2/RAS/MEK-ERK pathway in CRC cells. Further investigations using protein co-immunoprecipitation experiments could confirm the link between the integrin α1β1 subunit, Caveolin-1 and Grb2 in CRC. However, the RAS/RAF/MEK/ERK pathway could also be activated by other integrins including α2β1, in particular in the HT29 cell line [13]. Although herein the expression of the integrin α2 subunit did not increase in response to α1/*ITGA1* repression, a compensating role for α2β1 in this pathway cannot be excluded. Among the three cell lines we used for this study, HT29 contains a BRAF mutation, SW480 has a mutated KRAS, whereas the T84 cell line does not have a mutation in the RAS/MEK/ERK pathway [29]. This could explain why proliferation was more highly affected in the T84 cell line compared to the two other cell lines. Our results suggest that despite the presence of mutated KRAS and BRAF, in colorectal cell lines, the presence of α1β1 upstream of these signal transductors remains required at least for cell proliferation. This observation is consistent with a previous study showing that this integrin remains important for lung cancer cells even with the KRAS mutation [16]. In fact, the KRAS mutation has been reported to be present in 50% of colorectal cancers [30,31] and is often mono-allelic [32], suggesting the presence of mutated and non-mutated KRAS proteins in the same cells. It is noteworthy that KRAS functions as an on/off system: active state when binding to GTP (guanosine triphosphate) and inactive when GTP is hydrolyzed to GDP (guanosine diphosphate) by a GAP (GTP as activating proteins) [33]. The most frequent KRAS G12D mutation prevents the action of the GAP enzyme, which makes it constitutively active, while on the other hand, the ratio between the number of mutated and non-mutated KRAS in each cell determines the constitutively active state of the RAS/MEK/ERK pathway, like a rheostat [33]. In addition, KRAS cannot be functional if it is not recruited by its farnesyl group to the cytoplasmic membrane, even though it is constitutively active. Furthermore, the localization of KRAS may depend on the organization of the actin cytoskeleton, which is regulated by the integrins upstream [34,35]. Such an action of the integrin α1β1 still needs to be demonstrated.

Regarding the contribution of the α1β1 integrin to cell survival, our results show that repression of the expression of the integrin α1 subunit/*ITGA1* increases apoptosis only in non-adherent cells. In fact in the normal intestinal epithelium, the involvement of integrins (mainly β1) in cell survival depends on the state of cell differentiation, the integrin involved, the recruitment of focal adhesion kinase (FAK) and/or SRC and also on the activation of the phosphatidylinositol-3 kinase (PI3-K)\AKT8 virus oncogene cellular homolog (Akt) and/or MEK/ERK survival pathways, in addition to the contribution of growth factor receptors such as the epidermal growth factor receptor [20,36,37]. Indeed, neutralizing α2β1 and α5β1 integrins renders undifferentiated intestinal cells sensitive to anoikis, while neutralization of the α3β1 integrin increases the anoikis of differentiated cells [20]. This last integrin is not expressed at significant levels in colorectal cancer cells [38,39]. On the other hand, integrin α6β4 has no effect on the apoptosis of CRC cells, whereas expression of integrin α8β1 in colorectal cancer cells makes them susceptible to anoikis [40]. This integrin is not normally expressed by CRC cells [40], in contrast to integrins α1β1 and α2β1. As mentioned above, the fact that the expression of α2β1 was not increased in any of the three cell lines does not rule out the possibility of the recruitment of α2β1 to partially compensate for the loss of signalization resulting from α1β1 repression, especially for adherent cells because α2β1 can regulate the MEK/ERK pathway as well as the phosphorylation of p38 by distinct signaling pathways [41]. Incidentally, it should be noted that inhibition of the MEK/ERK pathway induces apoptosis for both T84 and HT29 [37].

Integrins are major actors in the migration and invasion of tumor cells [19,42] as well as in resistance to anoikis [3], which are determining steps for metastasis. Integrin α1β1 is involved in the migration of leukocytes and also tumor cells of lung and mammary cancers [7,43], which matches our observations in CRC. This integrin is important for the migration of HT29 and T84 cells, whereas SW480 cells have not been tested for this function because they are sensitive to treatment with hydroxyurea, as previously reported [21]. It should be noted that in the murine model, integrin α1β1 is also involved in tumor invasion in hepatocellular carcinoma, pulmonary and mammary carcinoma, through regulation of stromelysin-1 expression [44,45]. In CRC, a previous study showed that integrin α1β1 participates in the invasion of colorectal cancer cells by recruitment of the FAK/SRC complex and activation of the p130/Cas/Janus kinase pathway and subsequently by the increased expression of matrix metalloproteinases 2 and 9 [9].

In mouse models, abolition of integrin α1 subunit expression resulted in smaller and fewer lung tumors despite the presence of an active KRAS mutation [16]. These mice have prolonged survival compared to control mice. These tumors have decreased carcinomatous cell proliferation with reduced ERK1/2 phosphorylation associated with increased in vivo and in vitro apoptosis [16]. Our findings in tumors derived from HT29 and T84 cells are consistent with these results. The fact that the knockdown of the integrin α1 subunit/*ITGA1* resulted in apparently smaller cells for the T84 but not the HT29 cell line could be an indication that α1β1 also plays a role in cell shape and/or differentiation. In fact, previous reports have indicated that T84 cells spontaneously polarize/differentiate in culture forming tight junctions and brush borders, whereas HT29 cells remain unpolarised under standard culture conditions [46,47]. While α1β1 was predominantly observed in the basolateral domain of undifferentiated cells of the normal crypt of the small intestine and the colon [11,12,48], the knockdown of the integrin α1 subunit/*ITGA1* was found to cause alteration at the level of the tight junctions in intestinal cells [49] suggesting a potential role on cell polarization. 

The presence of extensive necrosis in sh-ITGA1 derived tumors, which is often at the center of the tumors, suggests that this necrosis is secondary to the reduction in vascularization of these tumors as observed herein, which could be related to integrin α1 subunit/*ITGA1* repression in tumor cells. Indeed, integrin α1β1 appears to be important for endothelial cell proliferation and tubulogenesis [6]. In this study, selective residue mutations in the α1 cytoplasmic tail showed that the above functions are dependent on the α1 subunit [6]. For instance, the Pro1142 and Leu1145 appear to be required for extracellular signal-regulated kinase (ERK) activation and cell proliferation [6]. Other studies have shown that neutralizing α1β1 function in mice results in a decrease in intra-tumor vasculature [4,7,50]. Furthermore, it has been reported that intra-tumoral endothelial cells can be derived from tumor cells [51]. These cells have been described as containing Weibel palade bodies as in endothelial cells and epithelial markers (cytokeratin 7) at the same time [52]. Although more study is needed to explain the presence of fewer vessels in the tumors, one may speculate that the reduction in these tumor-derived endothelial cells is related to the integrin α1 subunit/*ITGA1* repression in the parental tumor cells.

Taken together, our findings demonstrate the promoting role of the α1β1 integrin in CRC progression, by regulating cell proliferation, survival and migration. This is in concert with our previous finding that the expression of this integrin is increased by the MYC oncogenic factor [12]. It should be noted that a positive regulatory loop mechanism between MYC and integrin α1β1 is possible and could involve other integrins regulated by MYC, including α6β4 [53]. Indeed, if the RAS/MEK/ERK pathway is activated by α1β1, this could contribute to the stabilization of MYC by its phosphorylation by ERK, and MYC in turn would induce higher expression of the ITGA1 subunit. This association between MYC and its targets including integrin α1β1 may constitute one of the promising combined therapeutics in the treatment of CRC.

## 4. Materials and Methods

### 4.1. Cell Culture and Lentivirus-Mediated RNA Interference

Colorectal cancer cell lines T84, HT29 and SW480 were initially obtained from the American Type Culture Collection (Manassas, VA, USA). T84 cells were cultured in Dulbecco’s modified Eagle’s medium/F12 (Life Technologies, Burlington, ON, Canada) with 5% fetal bovine serum, 2.5 mM GlutaMAX, 15 mM HEPES and 0.5 mM pyruvate. HT29 and SW480 cells were cultured in Dulbecco’s modified Eagle’s medium (Life Technologies), supplemented with 10% fetal bovine serum, 2 mM GlutaMAX and 10 mM HEPES. All cells were maintained in a 5% CO2-humidified atmosphere at 37°C and were verified for the absence of mycoplasma contamination. Lentivirus sequences, containing short hairpin RNAs targeting *ITGA1* obtained from Sigma-Aldrich, were: sh-ITGA1; 5′-CCGGCCCACATTTCAAGTCGTGAATCTCGAGATTCACGACTTGAAATGTGGGTTTTTG-3′ another sh-ITGA1 (identified sequence B) was also used in preliminary studies; 5′-CCGGGTGGGCATCAAAGACGTGTTTCTCGAGAAACACGTCTTTGATGCCCACTTTTTG-3′ and sh-ctl; 5′CCGGGTGGGCATCAAAGACGTGTTTCTCGAGAAACACGTCTTTGATGCCCACTTTTTG-3′.

All viruses were prepared in HEK293T cells as previously described [12]. HT29, SW480 and T84 cells, at 60% confluence, were infected for 48 h and then selected with puromycin (2.5–10 μg/mL) for 10 days.

### 4.2. Western Blot Analysis

Proteins were extracted from cell and xenograft tumors and processed for SDS-polyacrylamide gels electrophoresis and Western blot analysis as described previously [12]. Primary antibodies used were anti-human integrin α1/CD49A (1:2000, AF5676, R&D System, Minneapolis, MN, USA), anti-human integrin α2 (1:10,000, Ab 133757, Abcam, Toronto, ON, Canada) and anti-β-actin (1:80,000, C4, Millipore, Etobicoke, ON, Canada). Secondary antibodies were horseradish peroxidase-conjugated anti-sheep (12-342, Millipore), anti-rabbit (NA934V, Amersham, Mississauga, ON, Canada) and anti-mouse (NA913V, Amersham) antibodies. All experiments were repeated a minimum of three times.

### 4.3. Growth Curve Assay

T84, HT29 and SW480 cells were seeded in 12-well plates at 5 × 10^4^ cells/dish in their respective media. Cell number was counted using a TC10 Automated Cell Counter (Bio-Rad, Saint-Laurent, QC, Canada).

### 4.4. Cell Proliferation Assay

Proliferation assays were carried out using 5-bromo-2-deoxyuridine (BrdU) incorporation following the manufacturer’s instructions (Roche, Laval, Quebec) and the procedure previously published [54].

### 4.5. Migration Assay

Cells were seeded at high density in 60 mm dishes. Twenty-four hours before the scratch assay, cells were treated with hydroxyurea 2 mM (Sigma). The wound was performed using a P200 pipette tip, and immediately, the dishes were washed with PBS three times before adding fresh medium containing hydroxyurea and 20 µg/mL of type I collagen (BD Biosciences Pharmingen, Mississauga, ON, Canada). The wound healing was monitored at 0, 6, 12, 24 and 48 h. Micrographs were taken, in marked areas, using an adapted camera (RS Photometrics CoolSNAP, Tucson, AZ, USA). The quantification of the evolution of the healing was analysed with Metamorph software (Metamorph Imaging System, Molecular Devices, Sunnyvale, CA, USA).

### 4.6. Anoikis Assays

To induce anoikis, cells were kept in suspension for 24 h in serum-free medium containing 5–10 µg/mL of puromycin, as previously described [55]. Briefly, cells were trypsinized and then immediately seeded onto poly-2-hydroxyethyl methacrylate coated dishes. Twenty-four hours later, cells in suspension were collected and suspended in radioimmunoprecipitation assay (RIPA) buffer [Tris HCl pH 8.0 50 mM, NaCl 150 mM, dithiothreitol 1 mM, ethylenediaminetetraacetic acid (EDTA) 0.5 M, NP-40 0.5%, Na deoxycholate acid 0.5%, SDS 0.1%, Na vanadate 100 µM, phenylmethylsulfonyl fluoride 1 mM in isopropanol, β-glycerol phosphate 40 mM, 1 tablet of protease inhibitors (Roche)] without p-nitrophenyl phosphate for protein extraction.

### 4.7. Caspase 3 Activity Assays

Fluorometric quantification of caspase activity using acetyl-Asp-Glu-Val-Asp 7-amino-4-methylcoumarin (Ac-DEVD-AMC; Calbiochem) as a substrate for caspase 3 was performed as previously described [20]. Reactions were read with a Hitachi S-2500 Spectrofluorometer. Caspase activity was expressed as arbitrary units, which were calculated for each cell line as fold change compared to the control condition.

### 4.8. Xenografts and Tumor Immunohistochemical Staining

To investigate the involvement of the integrin α1β1 in the tumorigenicity of colorectal cancer cells, 5–6 week-old athymic female CD1 mice were purchased from Charles River (Wilmington, MA, USA). The experimental protocol used was approved by the Ethics Committee for Animal Experimentation of the Université de Sherbrooke. For each cell line, T84 or HT29, an amount of 2 × 10^6^ of sh-ITGA1 or sh-ctl cells was gently injected into the dorsal subcutaneous tissue of mice. The size of the tumors was monitored and recorded twice a week. The tumor volume was calculated using the formula V = (D × d2)/2, where D is the length (largest tumor diameter) and d represents the width or the perpendicular tumor diameter. Tumors were monitored and their size was measured frequently during 50 to 60 days, until the mice were sacrificed. The tumors were removed and cut into two pieces along the major axis. One portion was cut into small pieces and then disrupted in Laemmli buffer using a polytron, followed by standard protein extraction as described previously [12]. The other portion was processed for histological analysis. Samples were fixed in paraformaldehyde overnight, washed in 70% ethanol and then kept at 4 °C until processed for paraffin embedding. Blocks were then cut into 5 µm sections and stained with hematoxylin and eosin. Additional slides where used for the immunohistochemical staining procedure, as described previously [11]. Primary antibodies were: anti-human integrin α1 purified polyclonal sheep IgG (5 μg/mL, AF5676, R&D Systems, Minneapolis, MN, USA) for the detection of the α1 integrin subunit, and anti-CD31 (5 μg/mL, the P2B1 monoclonal antibody developed by E.A. Wayner and G. Vercellotti and was obtained from the Developmental Studies Hybridoma Bank, created by the NICHD of the NIH and maintained at The University of Iowa, Department of Biology, Iowa City, IA, USA) to detect blood vessels. The slides were scanned using a slide scanner (Nanozoomer 2.0-RS, Hamamatsu, Boston, MA, USA) and visualized using NDP.view2 software (NDP viewing software U12388-01, Hamamatsu).

## 5. Conclusions

In the present study, we show for the first time that the integrin α1β1 is important for regulating the proliferation of colorectal tumor cells as well as for their migration and survival. Moreover, using xenograft models, we observed that this integrin is important for the growth of colorectal tumors while its absence induces significant tumor necrosis. These findings provide new insights into understanding the implication of α1β1 integrin in colorectal carcinogenesis.
